# Retraction: RAB31 targeted by MiR-30c-2-3p regulates the GLI1 signaling pathway, affecting gastric cancer cell proliferation and apoptosis

**DOI:** 10.3389/fonc.2025.1713927

**Published:** 2025-10-03

**Authors:** Frontiers Editorial Office

The journal retracts the 26 November 2018 article cited above.

Following publication, concerns were raised regarding the integrity of the images in the published figures. The authors failed to provide the raw data or a satisfactory explanation during the investigation, which was conducted in accordance with Frontiers’ policies. Given the concerns about the validity of the data, and the lack of raw data, the editors no longer have confidence in the findings presented in the article.

This retraction was approved by the Chief Editors of Frontiers in Oncology and the Chief Executive Editor of Frontiers. The authors received a communication regarding the retraction and had a chance to respond. This communication has been recorded by the publisher.

